# Outcomes and practice patterns with hemodiafiltration in Shanghai: a longitudinal cohort study

**DOI:** 10.1186/s12882-019-1219-z

**Published:** 2019-02-01

**Authors:** Weiming Zhang, Changlin Mei, Nan Chen, Xiaoqiang Ding, Zhaohui Ni, Chuanming Hao, Jinghong Zhang, Jinyuan Zhang, Niansong Wang, Gengru Jiang, Zhiyong Guo, Chen Yu, Yueyi Deng, Haiming Li, Qiang Yao, Mark R. Marshall, Martin J. Wolley, Jiaqi Qian

**Affiliations:** 1 Department of Nephrology, Renji Hospital, School of Medicine, Shanghai Jiao Tong University, Shanghai, 200127 China; 2 Department of Nephrology, Changzheng Hospital, Second Military Medical University, Shanghai, 200003 China; 3 Department of Nephrology, Ruijin Hospital, School of Medicine, Shanghai Jiao Tong University, Shanghai, 200025 China; 4 Division of Nephrology, Zhongshan Hospital, Shanghai Medical College, Fudan University, Shanghai, 200032 China; 5 Division of Nephrology, Huashan Hospital, Shanghai Medical College, Fudan University, Shanghai, 200040 China; 6 Department of Nephrology, 85 Hospital of People’s Liberation Army, Shanghai, 200052 China; 7 Department of Nephrology, 455 Hospital of People’s Liberation Army, Shanghai, 200052 China; 8 Department of Nephrology, Shanghai Sixth People’s Hospital, Shanghai Jiao Tong University, Shanghai, 200233 China; 9 Department of Nephrology, Xinhua Hospital, School of Medicine, Shanghai Jiao Tong University, Shanghai, 202150 China; 10 Department of Nephrology, Changhai Hospital, Second Military Medical University, Shanghai, Shanghai, 200433 China; 11 Department of Nephrology, Tongji Hospital, Tongji University, Shanghai, 200092 China; 12 Department of Nephrology, Longhua Hospital, Shanghai University of Traditional Chinese Medicine, Shanghai, 200032 China; 13 Baxter China Investment Co Ltd, Medical Affairs, Shanghai, 200031 China; 14 Baxter Healthcare (Asia) Pte Ltd, Medical Affairs, Singapore, 189673 Singapore; 15 School of Medicine, Faculty of Medical and Health Sciences, University of Auckland, Auckland, 1142 New Zealand; 16 Department of Renal Medicine, Counties Manukau Health, Auckland, 1640 New Zealand; 17 Department of Renal Medicine, Royal Brisbane and Women’s Hospital, Brisbane, Queensland 4029 Australia; 18 School of Medicine, University of Queensland, Brisbane, Queensland 4072 Australia

**Keywords:** China, Hemodiafiltration, Epidemiology, Mortality, Hemodialysis

## Abstract

**Background:**

Globally, there is increased clinical interest and uptake of hemodiafiltration (HDF) for increased removal of uremic toxins. To date, there has been no epidemiological analysis of HDF in China. We present HDF practice patterns and associated mortality risk in Shanghai.

**Methods:**

This is an observational, prospectively collected, retrospective analysis of 9351 Chinese patients initiating hemodialysis in Shanghai from 2007 to 2014. The primary exposure was hemodialysis sub-modality at inception, classified into hemodiafiltration (HDF) and hemodialysis (HD), with adjustment for concommitant hemoperfusion. The primary outcome was patient mortality. We used Cox proportional hazards regression and Fine and Gray’s proportional subhazards regression, with multiple imputation of missing co-variates by the chained equation method, adjusting for demographic and clinical variables.

**Results:**

Overall, patients in the cohort were younger, with a more males, and with a lower body mass index when compared to corresponding non-Asian cohorts. Mortality rate was low although it doubled over the period of observation. HDF utilization increased from 7% of patients in 2007 to 42% of patients in 2014. The majority of patients received HDF once a week. The adjusted hazard ratio of death (95% confidence intervals) for HDF versus HD was 0.85 (0.71–1.03), and corresponding sub-hazard ratio 0.86 (0.71–1.03). There was strong effect modification by age. In those aged 40–60 years, the hazard ratio (95% confidence intervals) was 0.65 (0.45–0.94), and sub-hazard ratio also 0.65 (0.45–0.95).

**Conclusions:**

Our study has certain limitations resulting from the limited number of co-variates available for modelling, missing data for some co-variates, and the lack of verification of data against source documentation. Notwithstanding, there is evidence of clinical benefit from HDF in China, and potential to improve patient outcomes through the greater removal of middle and larger uremic solutes.

**Electronic supplementary material:**

The online version of this article (10.1186/s12882-019-1219-z) contains supplementary material, which is available to authorized users.

## Background

China has one of the largest – if not the largest - chronic kidney disease (CKD) populations on the globe [1]. According to the latest report from the Chinese Renal Data System [2], there were 447,435 prevalent patients on hemodialysis (HD) and 74,138 on peritoneal dialysis (PD) at the end of 2016 (https://www.cnrds.net). There has been rapid growth in dialysis for several reasons. Firstly, there is an increasing prevalence of risk factors for progressive CKD, most notably diabetes mellitus and increased body size [3–5]. Secondly, coverage for dialysis via social insurance has expanded markedly over the last 5 years, and dialysis is increasingly offered to patients. Thirdly, there is increasing health literacy amongst healthcare consumers in developed areas of China, who have grown accustomed to advanced standards in healthcare, especially in larger cities. Finally, the largest rural to urban migration in human history has led to generally better access to health services and higher incomes for most people [6].

Globally, there is increased clinical interest and uptake of therapies that have increased removal of uremic toxins relative to high flux HD. The currently available methods for doing so include more intensive HD [7, 8], leakier dialyzer membranes [9, 10], or greater convective clearance [11–19]. At the present time, greater convective clearance using hemodiafiltration (HDF) is the most commonly applied means for extending uremic solute clearance in routine clinical practice. To date, there has been no epidemiological analysis of HDF in China. In this article, we describe and analyses the evolving practice patterns and outcomes of HDF in China using the Shanghai Renal Registry (SRR), which was begun in 1996 by the Shanghai Society of Nephrology and Shanghai Center for Hemodialysis Quality Control (http://sh.cnrds.org). The registry prospectively collects data on all patients treated with maintenance renal replacement therapy in that city, and at the time of this study covered all 66 dialysis providers. We report on HDF experience in a large cohort of Chinese patients initiating HD in Shanghai from 2007 to 2014.

## Methods

### Study design

We performed an retrospective observational cohort study using an intention-to-treat framework [20]. None of the authors of the manuscript had access to any information that could be used to identify individual participants or their treating center during or after data collection. The Ethics Committee of the Shanghai Clinical Research Center (http://www.scrcnet.org/IEC_en.asp) reviewed and approved the study design and execution, and waived the need for individual consent to participate on the basis of the research being a non-interventional study with unidentified data.

### Participants and data source

The SRR is a government-mandated registry that collects information on all treated end-stage kidney disease (ESKD) patients from all dialysis centers in Shanghai, China. The SRR began as the Shanghai Dialysis Registry in 1996 [21], and includes those treated with all forms of renal replacement therapy (RRT) including transplantation, followed from renal replacement inception until death or loss to follow-up. The SRR excludes those with acute kidney injury, and defines treated ESKD patients as those for whom dialysis is intended to be indefinite, and offers general guidance that patients with a treatment period of less than 90 days should not be included. Minimum data collected for the registry includes the centre, patient demographics, exact dates for dialysis inception, each change of renal replacement modality, and loss to follow-up or death. Discretionary data include details of patient co-morbidity, biochemical and laboratory tests, details of renal replacement regimens, and medications. Data are updated quarterly via an online user interface, and facility specific reports provided on an annual basis. Structure and methods of the registry have been reported elsewhere [22, 23].

From the larger dataset, we created a cohort of incident adult patients (aged > = 18 years) who initiated RRT as either inpatients or outpatients between January 1, 2007 and December 31, 2014 in Shanghai, China. This inception cohort was restricted to only those initiating RRT with HD, HDF, or hemoperfusion (HP). Patients were followed up until death, dropout, return of renal function, transfer out of the SRR network, permanent switch to PD (defined as more than 30 days of continuous treatment), kidney transplantation or December 31, 2014, whichever occurred first.

### Exposure variables

The primary exposure was treatment with HDF. We adjusted for patient-related factors using the first recorded observations for each patient. These data are intended to reflect status at RRT inception or after a short period of stabilization, although the SRR network does not specify collection period or procedures. The following data were included: age, gender, primary kidney disease as recorded in the SRR (primary nephropathies [glomerulonephritis], secondary nephropathies [diabetes, hypertension, systemic diseases with renal manifestations], and other causes [urinary tract infection (UTI) / stones / urological / malignancy]), pre-dialysis weight, body mass index (BMI), serum albumin, serum (unadjusted) calcium, serum phosphate, total cholesterol, estimated glomerular filtration rate (eGFR) by the abbreviated Modification of Diet in Renal Disease (MDRD) formula for Chinese [24], creatinine index from Canaud et al. [25], and year of dialysis inception.

We also adjusted for treatment-related factors also using the first recorded observations for each patient. These included: interdialytic weight gain (as a % of pre-dialysis weight), RRT frequency (per week), dialysis dose (single pool Kt/V per treatment), vascular access (arteriovenous fistula/graft, central venous catheter [CVC] /other), and the application of HP (e.g such as using a neutral macroporous resin apparatus [26]).

Continuous or ordinal co-variates other than age were modeled as clinically relevant quantiles in order to avoid the assumption of linear relationships.

### Outcome variable

The primary outcome was death on HD. The recorded outcome of “withdrawal from dialysis” was modelled as death. We assessed switch to PD, kidney transplantation, transfer out of the SRR network and loss to follow-up for unrecorded reasons as competing risks, since patients reaching these endpoints were no longer at risk of dying while being on HD.

### Statistical methods

For the primary analysis, we firstly constructed models for survival using Cox proportional hazards regression, censoring for all competing risks such as switch to PD, kidney transplantation, transfer out of the SRR network and loss to follow-up for unrecorded reasons. In such statistical models, probabilities of death in censored patients are still modelled - their deaths are calculated as happening at a time after the competing event, with the same probability (conditional on covariates) as those who had remained on HD and already died.

To more carefully account for competing risks, we also constructed models using Fine and Gray’s proportional subhazards model, where switch to PD and kidney transplantation were not censored but modelled as competing risks [27]. In this model and the Cox proportional hazards model, we censored patients for transfer out of the SRR network and loss to follow-up for unrecorded reasons, under the assumption of independent and non-informative censoring.

The SRR is intended to record only those patients with end-stage kidney disease, and not those with acute kidney injury. We could not be sure about adherence to this guideline by contributing HD units, however, and we therefore performed supplementary analyses in a restricted dataset excluding those with less than 90 days on dialysis.

We removed covariates from the model using a backward stepwise process starting with the covariate with the highest *P* value from two-tailed Wald tests of the individual coefficients, using the partial likelihood ratio test to compare the new model with the older one. We selected confounders for the final model according to both biological plausibility and comprehensibility, aiming for the most parsimonious model based upon the significance of the covariate within the model as assessed by the two-tailed partial likelihood ratio test *P* value at a level of 0.2 when jointly adjusted for other covariates.

We examine effect modification by era, serum albumin, and patient age using two-way interaction terms in the main-effects models. These interactions were chosen as being clinically plausible, as indicated by both published literature as well as the cumulative clinical experience of the research team. The significance of interaction terms were assessed by the two-tailed partial likelihood ratio test *P* value at a level of < 0.05 when jointly adjusted for other covariates.

We tested the proportional hazards assumption quantitatively using scaled Schoenfeld residuals, and qualitatively by using -ln [−ln(survival)] versus ln(analysis time) plots and comparing the goodness of fit between plots of Kaplan-Meier observed survival curves to the corresponding curves predicted by Cox models.

The data used for this study had important degrees of missingness. We examined missingness of other covariates using logistic regression. Missingness was found to depend on observed data on regression analysis, implying a “missing at random” (MAR) rather than “missing completely at random” (MCAR) data structure. We therefore imputed missing values to avoid distorted inference from complete case analysis in a non-representative sample of the study population. For imputation, we used the chained equation method based on iterative multiple regression models with all other variables included (with categorical covariates expanded as indicator variables) [28, 29]. We included all patient-related covariates, treatment-related covariates, and the dependent variable (primary and competing outcomes, loss to follow-up/transfer) as co-variates in imputation models [29, 30], and constrained imputation of missing variables to the observable data range using truncated regression. We ran imputation models separately for each variable before full imputation, to test for convergence and misspecification, and to assess for interactions [29].

For all imputation, we included potential interactions between the imputed variables as covariate terms, thus avoiding imputed values reflecting only linear relationships. We imputed within different subsamples for era of inception and renal replacement treatment frequency, to preserve higher-order dependencies for these variables. We imputed 20 data sets to reduce sampling variability from the imputation process [31, 32], and combined results of analyses using Rubin’s rules [33]. This number of imputations was guided by inspection of Monte Carlo errors, and the achievement of what has been suggested to be an acceptable amount of error 1) the error for a coefficient is less than or equal to 10% the coefficient’s standard error 2) the error of a coefficient’s T-statistic is less than or equal to 0.1, and 3) the error of a coefficient’s *P*-value is less than or equal to 0.01 if the true *P*-value is 0.05, or less than or equal to 0.02 if the true *P*-value is 0.1 [29].

Where necessary, we made comparisons between non-imputed groups using the Fisher’s exact and Wilcoxon rank sum test, and comparisons involving imputed groups using linear or quantile regression [34]. We computed effect size statistics for non-imputed groups only using Cohen’s d for continuous variables and Cohen’s ω for categorical ones [35]. Statistical significance was attributed to associations if the two-tailed *P* value was < 0.05.

Analyses were performed using Stata Intercooled MP/14.2 (StataCorp, www.stata.com).

## Results

### Participants and outcome data

The inception cohort contained 14,941 patients with complete primary exposure and outcomes data. Of these, there were 5590 patients without complete data for outcomes, age, gender, primary kidney disease, and renal replacement modality / dialysis characteristics. These patients were excluded from further analysis, leaving 9351 with complete data for the above variables. The excluded and included datasets are shown in Additional file 1: Table S1 (available as online supplementary material), along with their respective degrees of missingness. There were some minor differences between the excluded and included datasets, although all differences were small or very small in terms of effect size.

Of the 9351 patients in the included dataset, 4201 had no missing covariate data at all. The other 5150 required imputation for some degree of missingness for covariates other than the ones mentioned above (complete case and imputed variables shown in Additional file 2, available as online supplementary material). The final dataset for analysis consisted of 9351 patients over 29,250 patient-years, in which 982 patients died, 259 underwent kidney transplants, 240 changed to PD, and 7870 patients were censored (5484 because of end of follow-up, 64 for return of renal function, 2294 for transfer out to the SRR network, and 28 for loss to follow-up).

**Fig. 1:**
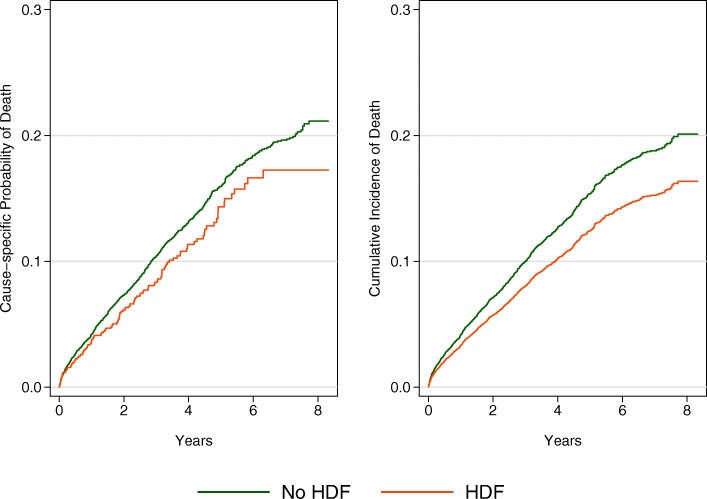
Cause-specific probability (from Kaplan Meier estimates) and cumulative incidence (from the competing risks regression) of death

The annual mortality rate (95% confidence interval) was 3.4 per 100 pt./years (3.2–3.6) overall, although this increased rapidly over the period of observation. Figure 1 shows the probability (from Kaplan Meier estimates) and cumulative incidence (from competing risks regression) of death over the follow up.

### Descriptive data

Table 1 summarizes the clinical characteristics of the final dataset, and also compares these characteristics by HDF versus HD. The following key observations can be made.

**Table 1:** Patient characteristics at renal replacement therapy (RRT) inception, by era. All results are presented as median (interquartile range) or n(%)

		All	No HDF	HDF
N		9351	7207	2144
Age (years)*		58 (47, 71)	59 (47, 72)	56 (44, 67)
Gender*	Male	5613 (60%)	4247 (59%)	1366 (64%)
	Female	3738 (40%)	2960 (41%)	778 (36%)
Cause of ESKF	Primary	3892 (42%)	2969 (41%)	923 (43%)
	Secondary	3472 (37%)	2714 (38%)	758 (35%)
	Other	1987 (21%)	1524 (21%)	463 (22%)
BMI (kg/m^2^)*		22.2 (20.0, 24.8)	22.2 (20, 24.7)	22.5 (20.3, 25.0)
Weight (kg)*		61.6 (53.8, 69.7)	61.1 (53.5, 69.0)	63.2 (54.9, 71.8)
Height (m)*		1.67 (1.60, 1.72)	1.66 (1.60, 1.72)	1.68 (1.60, 1.73)
Hemoglobin (g/L)	All patients	97.1 (81.9, 111.0)	97.1 (81.5, 111.0)	97.3 (82.0, 111.1)
	> = 3 x week RRT	97 (81, 111)	97 (81, 111)	97 (81, 111)
	> = 3 x week RRT & Kt/V > =1.2	98.2 (82.4, 111.9)	98.1 (82.2, 111.8)	96.5 (83, 112.1)
eGFR (mL/min/1.73m^2^)	All patients	6.0 (4.5, 8.2)	6.0 (4.5, 8.3)	6.0 (4.5, 8.1)
	> = 3 x week RRT	6.1 (4.6, 8.5)	6.2 (4.6, 8.6)	6.0 (4.5, 8.2)
	> = 3 x week RRT & Kt/V > =1.2	6.3 (4.7, 8.6)	6.4 (4.7, 8.7)	6.2 (4.6, 8.3)
Creatinine Index (mg/kg/day)	All patients*	21.2 (19.0, 23.7)	21.2 (18.9, 23.6)	21.5 (19.3, 23.9)
	> = 3 x week RRT*	21.2 (18.9, 23.5)	21.0 (18.8, 23.4)	21.4 (19.2, 23.8)
	> = 3 x week RRT & Kt/V > =1.2*	21.1 (18.9, 23.5)	20.9 (18.8, 23.3)	21.5 (19.3, 23.7)
Albumin (g/L)	All patients*	36 (31.8, 40.2)	36 (31.5, 40)	36.8 (32, 40.9)
	> = 3 x week RRT*	36 (31, 40)	36 (31, 40)	36.5 (32, 40.7)
	> = 3 x week RRT & Kt/V > =1.2*	36.8 (32, 40.8)	36.4 (32.0, 40.3)	37.1 (32.9, 41.0)
Total Cholesterol (mM)		4.0 (3.3, 4.8)	4.0 (3.3, 4.8)	4.0 (3.3, 4.8)
Unadjusted Calcium*		2.21 (2.05, 2.39)	2.21 (2.05, 2.39)	2.23 (2.06, 2.41)
PO4 (mM)	All patients	1.79 (1.40, 2.24)	1.79 (1.4,0 2.24)	1.77 (1.38, 2.21)
	> = 3 x week RRT	1.74 (1.37, 2.20)	1.75 (1.36, 2.20)	1.75 (1.38, 2
	> = 3 x week RRT & Kt/V > =1.2	1.76 (1.38, 2.2)	1.76 (1.37, 2.2)	1.77 (1.39, 2.22)
Frequency of RRT*	< 3 x week	2721 (29%)	2525 (35%)	196 (9%)
	> = 3 x week RRT	6630 (71%)	4682 (65%)	1948 (91%)
Kt/V	All patients*	1.29 (1.08, 1.53)	1.31 (1.10, 1.54)	1.24 (1.03, 1.47)
	> = 3 x week RRT*	1.26 (1.05, 1.48)	1.27 (1.06, 1.49)	1.24 (1.02, 1.47)
IDWG (% of weight)	All patients*	3.3 (2.0, 4.5)	3.4 (2.1, 4.6)	3.1 (1.7, 4.2)
	> = 3 x week RRT*	3.2 (1.8, 4.3)	3.2 (1.9, 4.4)	3 (1.7, 4.2)
Hemoperfusion*		165 (2%)	27 (0.5%)	138 (6%)
Vascular access*	AVF/AVG	4925 (53%)	3745 (52%)	1179 (55%)
	CVC / Other	4426 (47%)	3461 (48%)	965 (45%)

Abbreviations: *ESKF* end-stage kidney failure, *BMI* body mass index, *HDF* hemodiafiltration, *HP* hemoperfusion, *IDWG* inter-dialytic weight gain, *RRT* renal replacement therapy, *AVF* arteriovenous fistula, *AVG* arteriovenous (prosthetic bridge) graft; *CVC*, central venous catheter

*P < 0.05

Overall, the patients comprise a relatively young dialysis population, with a preponderance of males. Body size (weight, height and mass index) tended to be low compared to North American, European, or Australasian cohorts. Of note there are a relatively large number of patients on twice a week HD, a common feature of HD in China generally.

Patient characteristics differed significantly in those receiving HDF compared to those receiving no HDF. Those treated with HDF tended to have greater body size, were more likely to be males, more likely to be have a higher serum albumin, more likely to be on three-times a week therapy, and more likely to have an AV access.

**Fig. 2:**
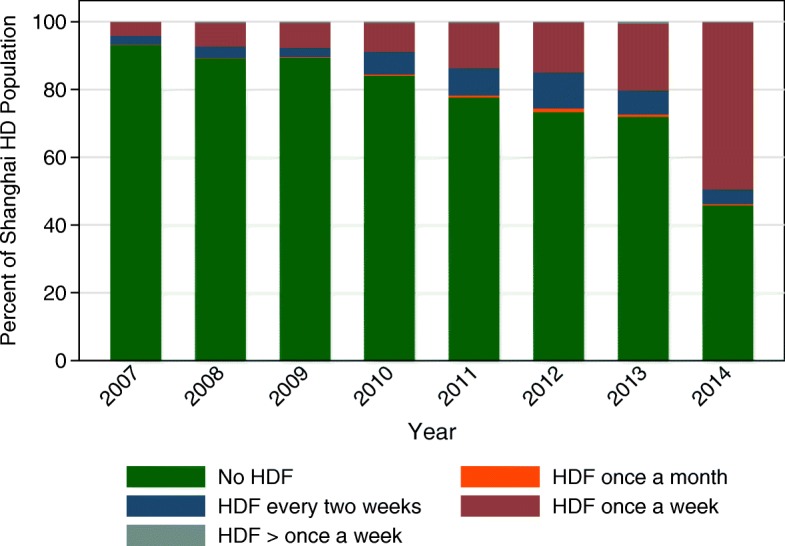
Change in utilization of hemodiafiltration (HDF) over time

**Fig. 3:**
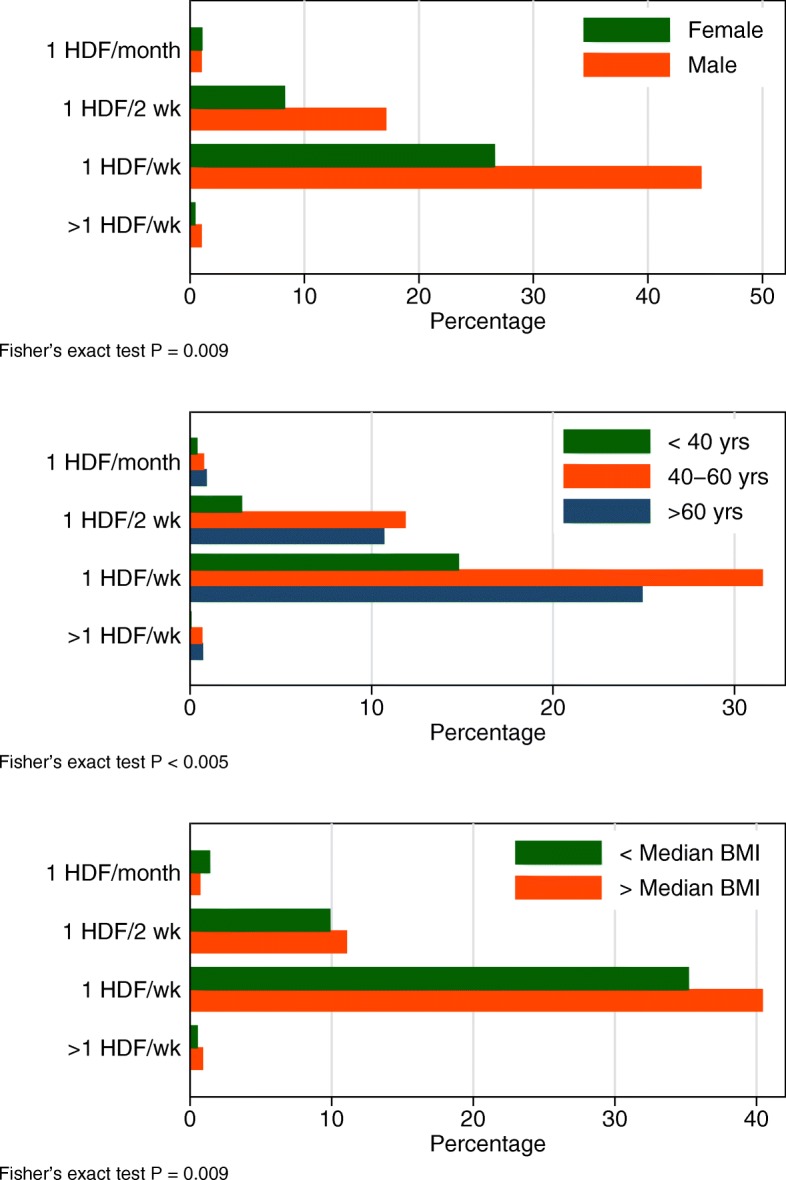
Hemodiafiltration (HDF) frequency of treatment, by gender, age, and body mass index (BMI)

The practice patterns around HDF also warrant comment. Overall 51 patients (0.5%) were treated with HDF once a month, 578 (6%) once every two weeks, 1585 (16%) once a week, and 34 (0.3%) twice or three times a week. Figure 2 illustrates the increase in HDF utilization by year, from 7% of patients in 2007 to 52% of patients in 2014. Overall, however, the application of HDF once a week remains the most common practice pattern over this period of observation. The distribution of the frequency of HDF varies by gender, age, and body size, indicating that HDF is applied slightly more frequently in younger, larger, males (Fig. 3).

### Main results

**Fig. 4:**
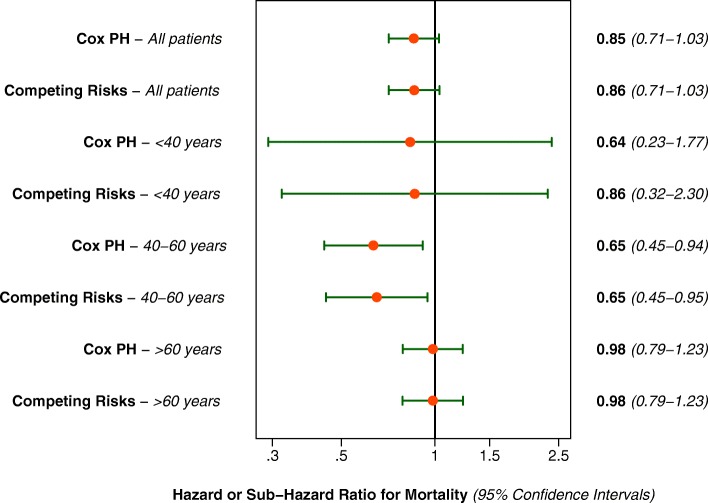
Main effects and subgroup model estimates for the effect of hemodiafiltration on mortality risk

The two primary main-effects models are summarized in Fig. 4, and shown in full in Additional file 3: Figures S1 and Additional file 4: Figure S2 (available as online supplementary material). There was reasonable convergent validity between two co-primary models, with only minor differences.

In the full models, increased patient mortality risk was associated with increasing age, male gender, hypoalbuminemia, anemia, CVC access, and era of dialysis inception, starting dialysis in the later part of the period of observation. Decreased mortality risk was associated with primary nephropathy as a cause of end stage kidney failure, and a trend to decreased mortality risk associated with lager body size, higher creatinine index, lower interdialytic weight gain, and less than thrice weekly renal replacement therapy. The covariates of Kt/V (*p* = 0.926, *p* = 0.651 in categories compared to reference category), blood purification using the neutral macroporous resin apparatus (*p* = 0.305 compared to no hemoperfusion), serum calcium (*p* = 0.993, *p* = 0.630 in categories compared to reference category), serum phosphate (*p* = 0.242, p = 0.630 in categories compared to reference category), and total cholesterol (*P* = 0.494 compared to reference category) were not associated with changes in mortality risk and were dropped from the model.

In terms of exposure of interest, HDF was associated with a trend to reduced mortality multivariable analyses, which fell short of being statistically significant at the 5% level. This trend was adjusted for other confounding, and independent of other risk factors for mortality included in Table 1 and also independent of era.

### Other results

There was no statistically significant interaction (*P* < 0.05) in the two primary models related to era, serum albumin, or frequency of renal replacement therapy, but a strong interaction by age (*P* = 0.0014). The interaction models are also summarized in Fig. 4. The effect modification by age is such that there is a trend towards improved mortality associated with HDF to improved mortality in those < 40 years of age, a statistically significant associated improvement in mortality risk in those 40–60 years, and no associated change in mortality risk in those greater than 60 years.

Supplementary analysis excluding those without 90 days of follow-up yielded similar estimates to the primary analyses - the adjusted hazard ratio (95% confidence interval) for mortality was 0.84 (0.68–1.03) using Cox proportional hazards regression, and the corresponding adjusted sub-hazard ratio (95% confidence interval) using competing risks regression was 0.84 (0.68–1.04).

## Discussion

There are four key findings in this study. The first is that HDF in China is most often applied once a week. There are two likely reasons for this practice pattern. The first is resource limitation – in the current China market, HDF machines are more expensive than HD machines, and HDF is not available in all facilities or to all patients within a given facility. The second concerns restrictions in reimbursement from social insurance, which is not universal in every part of China, with limited ability of many patients to self-pay for HDF.

The second key finding is that, even at a reduced frequency, HDF is independently associated with improved mortality risk in younger patients, and with a trend to improved outcomes in the population overall. This is directionally consistent with definitive evidence [11–19], and has not been reported before in the Chinese setting. Existing reports on HDF in China are limited to surrogate outcomes, most commonly clearance [36–58]. What reports there are, however, suggest that HDF in China is often applied with less than an optimal convective dose: blood flow rates range between 200 and 350 mL/min, dialysate flow between 500 and 800 mL/min, and convective dose in post-dilution mode of 15–25 L per treatment. In settings other than China, this has led to a paradoxical finding that HDF does not always lead to observable benefit in a “real world” setting [59]. In China, based on this study, this may not be the case.

The third finding is that mortality on HD in Shanghai is low. The annual mortality rate in our study for those initiating dialysis since 2010 was 5.4 per 100 pt./years, compared with 8.2 most recently in Europe [60], 9.8 in Japan [61], and 16.9 in the United States [62]. Data at hand do not allow detailed comparison with these other cohorts, although the mortality rate in our patients appears similar to those from the ongoing ChinaQ trial in China (NCT02378350, personal communication Xueqing Yu, Principal Investigator, November 2018), as well as another recent Chinese cohort study in which the 2010 annual mortality rate was reported as 7.7 per 100 pt./years [63, 64].

This low mortality rate is important in terms of external validity. In every population, surviving CKD to the point of dialysis selects hardy people [65], and this situation is exaggerated in our study. A decade or more ago, lack of reimbursement for dialysis in China resulted in markedly positive selection bias in dialysis patients. Anecdotally, these were affluent people who could afford dialysis, with a predicted longevity on dialysis that would justify dialysis in a resource-challenged healthcare setting. Over time, the increased access to care in China has led to a greater number of patients on HD; for example, in Shanghai the point prevalence of HD rose from 176 pmp in 2005 [66] to 380 pmp in 2014 [23]. This broadening of acceptance onto dialysis in Shanghai as well as elsewhere in China has led to patients with progressively less favourable prognoses, and is reflected by the decreasing survival on HD in Shanghai both in our study (Fig. S1), as well as in other Chinese cohorts [63, 64]. The relatively healthy nature of our cohort should be considered carefully, and the extent to which their response to therapy is more broadly applicable.

The final key finding in our study concerns the satisfactory outcomes with carefully applied utilization of < 3 x week RRT, which is consistent with other recent studies [67–70]. In China, resource constraints lead most nephrologists to delay initiation of RRT until there are clear clinical indications and/or symptoms of uraemia, and use < 3 x week RRT as much as possible. In our study, both higher eGFR and thrice weekly regimens at dialysis inception were both associated with increased mortality risk. This reflects customary practice in Shanghai - earlier and more intensive dialysis is reserved for sicker patients in an effort to preserve or improve health status, while the many healthier patients start only after the onset of clinical uraemia, in a carefully-monitored and incremental fashion.

Our study is an important one as it provides census-based and longitudinal approach. We can contrast the difference between our results and those of the recent Dialysis Outcome and Practice Patterns Study (DOPPS) data published as a cross-sectional study of prevalent patients across China [68]. Important differences include: Gender - 46% female in the DOPPS, 39% in this study; hemoglobin - 106 g/l in the DOPPS, 95.9 g/l in this study; frequency of RRT <3x/week - 26% in DOPPS, 21% in this study; AVF/AVG prevalence - 89.8% in the DOPPS, 46% in this study. These differences reflect differences between prevalent and cross-sectional (DOPPS) versus incident and longitudinal (SRR) cohorts.

Our study has three major weaknesses. The most important is residual confounding. Despite our efforts to account for differences between groups treated with HDF and HD, there is likely to be residual confounding that no amount of modelling can abrogate due to the non-availability of potentially important covariates. A large but unquantifiable part of the demonstrated association between HDF and better outcomes may therefore be from unmeasured differences between the cohorts. For instance, we do not have comprehensive information on medical co-morbidity such as the presence of diabetes mellitus, and no way for us to ensure balance between groups for characteristics such as these. As importantly, we do not have data on patient income status to identify financial advantage in one group or the other, and this may be favouring the HDF group and positively affecting mortality risk. As a result, our analyses cannot be considered conclusive, and the effect of HDF per se on the patient survival must still be a matter of speculation. In our study, there remains an unquantifiable but potentially high likelihood of important selection bias between groups as a result of unavailable covariates.

The second limitation is the significant amounts of missing data. In our study, 5590 out of 14,941 patients were excluded since they did have complete data for outcomes, age, gender, primary kidney disease, and renal replacement modality / dialysis characteristics. This ratio is not dissimilar to some other registry analyses [71], such as a recent United Kingdom renal registry analysis where ~ 5500 of 11,000 patients were excluded for missing data. The included patients in our study had complete data for these core variables (i.e. outcomes, age, gender, primary kidney disease, and renal replacement modality / dialysis characteristics), and acceptable amounts of missing data for imputation of other covariates. As acknowledged by others [71–76], the imputation of missing values of other variables is critical to avoid bias from complete case analysis with missingness that is not completely at random.

The third limitation is that the SRR (like all registries) is likely to be affected by significant ascertainment bias. Endpoints were recorded by electronic returns, but not validated against source documentation or other records.

## Conclusions

In conclusion, there is evidence of changing HD practice patterns in Shanghai, and increased HDF utilization. Our study suggests that the enhanced removal of middle sized and larger molecules improves outcomes in end-stage kidney failure populations from larger metropolitan centers, even when used within the resource constraints of the world’s most rapidly expanding and largest CKD population. The benefit of HDF is reasonably accepted to depend on convective dose [12], although it possible that the current standards for adequacy proposed from data in Europeans may be inappropriate for Chinese patients who are significantly smaller. Nonetheless, it is possible or even likely that even greater improvements might be seen in China with therapy that is applied in more standard fashion at three time a week with greater attention to convective dose.

## Additional files

Additional file 1: **Table S1.** Table of patient characteristics at renal replacement therapy (RRT) inception, comparing the included and excluded cohorts. (PDF 38 kb)

Additional file 2: Complete case and imputed variables (twenty imputations) used in the modelling. (PDF 149 kb)

Additional file 3: **Figure S1.** Full Cox proportional hazards (PH) main-effects model, fully adjusted for the main effects confounders listed in Table 1 (the marker represents point estimates, the whiskers, 95% confidence intervals). Abbreviations: HDF, hemodiafiltration; HB, hemoglobin; BMI, body mass index; eGFR, estimated glomerular filtration rate; RRT, renal replacement therapy; AV, arteriovenous; IDWG, inter-dialytic weight gain. (PDF 270 kb)

Additional file 4: **Figure S2.** Full competing risks main-effects model, fully adjusted for the main effects confounders listed in Table 1 (the marker represents point estimates, the whiskers, 95% confidence intervals). Abbreviations: HDF, hemodiafiltration; HB, hemoglobin; BMI, body mass index; eGFR, estimated glomerular filtration rate; RRT, renal replacement therapy; AV, arteriovenous; IDWG, inter-dialytic weight gain. (PDF 283 kb)

## Abbreviations

AVF: Arteriovenous fistula
AVG: Arteriovenous (prosthetic bridge) graft
BMI: Body mass index
CI: Confidence interval
CKD: Chronic kidney disease
CVC: Central venous catheter
DOPPS: Dialysis Outcome and Practice Patterns Study
eGFR: Estimated glomerular filtration rate
ESKD: End-stage kidney disease
HD: Hemodialysis
HDF: Hemodiafiltration
HP: Hemoperfusion
IDWG: Inter-dialytic weight gain
MAR: Missing at random
MCAR: Missing completely at random
MDRD: Modification of Diet in Renal Disease
PD: Peritoneal dialysis
PH: Proportional hazards
RRT: Renal replacement therapy
SRR: Shanghai Renal Registry
UTI: Urinary tract infection
