# Supplementary material for: Outcomes and practice patterns with hemodiafiltration in Shanghai: a longitudinal cohort study
Source: BMC Nephrol. 2019 Feb 1;20:34. doi: 10.1186/s12882-019-1219-z (PMC6359843; doi:10.1186/s12882-019-1219-z)
Supplement: Supplementary file 1 — Table S1. Table of patient characteristics at renal replacement therapy (RRT) inception, comparing the included and excluded cohorts. (PDF 38 kb) [file 12882_2019_1219_MOESM1_ESM.pdf]

Table S1.

Patient characteristics at renal replacement therapy (RRT) inception, comparing the included and excluded cohorts. All results are presented as median (interquartile range) or n(%).

		Excluded dataset	Included dataset	Cohen's d / Cohen's w	Missing data from excluded dataset	Missing data from included dataset
N		5,590	9,351			
Age (years)		58 (45, 71)	58 (47, 71)	not applicable	3,584 (64)	0 (0)
Gender*	Male	1,263 (63%)	5,613 (60%)	0.02	3,584 (64)	0 (0)
	Female	743 (37%)	3,738 (40%)			
Cause of ESKF*	Primary	625 (31%)	3,892 (42%)	0.06	3,584 (64)	0 (0)
	Secondary	1774 (36%)	3,472 (37%)			
	Other	536 (27%)	1,987 (21%)			
BMI (kg/m^2)		22.1 (20.0, 24.4)	22.2 (20.0, 24.7)	not applicable	4,897 (88)	2,811 (30)
Weight (kg)		61.8 (54, 68.9)	61.6 (53.8, 69.6)	not applicable	4,523 (81)	1,892 (20)
Height (m)		1.66 (1.60, 1.72)	1.67 (1.60, 1.72)	not applicable	4,897 (88)	2,813 (30)
Hemoglobin (g/L)		94 (79, 109)	98 (82, 111)	0.11	1,522 (27)	827 (9)
Creatinine (umol/L)		863 (661, 1088)	885 (692, 1096)	not applicable	4,906 (88)	3,518 (38)
Albumin (g/L)*		35 (31, 39)	36.2 (32.0, 40.3)	0.16	1,974 (35)	1,236 (13)
Total Cholesterol (mM)*		4.1 (3.4, 4.8)	4.0 (3.3, 4.7)	0.08	4,259 (76)	2,189 (23)
Unadjusted Calcium*		2.18 (2, 2.35)	2.21 (2.05, 2.39)	0.18	1,673 (30)	1,141 (12)
PO4 (mM)*		1.69 (1.35, 2.10)	1.78 (1.4, 2.23)	0.14	1,691 (30)	1,184 (13)
Frequency of RRT*	<3 x week	164 (3%)	2,721 (29%)	0.32	0 (0)	0 (0)
	>=3 x week RRT	5,426 (97%)	6,630 (71%)			
Kt/V		1.29 (1.11, 1.5)	1.3 (1.11, 1.54)	not applicable	4,947 (89)	3,999 (43)
IDWG (%)*		3.2 (1.9, 4.4)	3.4 (2, 4.5)	0.03	4,654 (83)	2,431 (26)
Hemodiafiltration		104 (20%)	2144 (23%)	not applicable	5,065 (91)	0 (0)
Hemoperfusion		17 (3%)	165 (2%)	0.02	5,065 (91)	0 (0)

Vascular access*	AVF/AVG	458 (38%)	4,653 (53%)	0.1	4,384 (78)	516 (6)
	Other	748 (62%)	4,182 (47%)			

Abbreviations: ESKF, end-stage kidney failure; BMI, body mass index; HDF, hemodiafiltration; HP, hemoperfusion; IDWG, inter-dialytic weight gain; RRT, renal replacement therapy; AVF, arteriovenous fistula; AVG, arteriovenous (prosthetic bridge) graft

* $P<0.05$
