# Supplementary material for: Outcomes and practice patterns with hemodiafiltration in Shanghai: a longitudinal cohort study
Source: BMC Nephrol. 2019 Feb 1;20:34. doi: 10.1186/s12882-019-1219-z (PMC6359843; doi:10.1186/s12882-019-1219-z)
Supplement: Supplementary file 2 — Complete case and imputed variables (twenty imputations) used in the modelling. (PDF 149 kb) [file 12882_2019_1219_MOESM2_ESM.pdf]

```
      name:  <unnamed>
       log:  /Users/markmarshall/Dropbox/SRRN/Manuscript/appendix1.smcl
  log type:  smcl
 opened on:  24 Jul 2015, 11:15:13
```

1 .
2 . mi xeq: summ bmi alb tot_chol totalcalcium po4 hb pre_d_wt creat KtV per

m=0 data:
-> **summ bmi alb tot_chol totalcalcium po4 hb pre_d_wt creat KtV per**

Variable	Obs	Mean	Std. Dev.	Min	Max
bmi	6540	22.54913	3.665202	10.47	46.02
alb	8115	35.86079	6.423378	11	57
tot_chol	7162	4.110973	1.151236	.56	15.01
totalcalcium	8210	2.227322	.2855613	1.07	3.47
po4	8167	1.869253	.6749525	.4	5.83
hb	8524	96.53156	21.78419	10	187
pre_d_wt	7459	62.42791	12.17227	21.3	138.4
creat	5833	903.7147	303.5485	104	2471
KtV	5352	1.338275	.3510049	.4	2.96
per	6920	3.27763	1.882586	-11.58392	11.20944

m=1 data:
-> **summ bmi alb tot_chol totalcalcium po4 hb pre_d_wt creat KtV per**

Variable	Obs	Mean	Std. Dev.	Min	Max
bmi	9351	22.556	3.663938	10.47	46.02
alb	9351	35.77099	6.38516	11	57
tot_chol	9351	4.106025	1.160547	.56	15.01
totalcalcium	9351	2.225304	.2835358	1.07	3.47
po4	9351	1.863746	.6719348	.4	5.83
hb	9351	96.29978	21.79526	10	187
pre_d_wt	9351	62.34796	12.1722	21.3	138.4
creat	9351	901.5938	306.7642	104	2471
KtV	9351	1.311041	.3484043	.4	2.96
per	9351	3.248563	1.894263	-11.58392	11.20944

m=2 data:
-> **summ bmi alb tot_chol totalcalcium po4 hb pre_d_wt creat KtV per**

Variable	Obs	Mean	Std. Dev.	Min	Max
bmi	9351	22.57145	3.667878	10.47	46.02
alb	9351	35.76925	6.440603	11	57
tot_chol	9351	4.115149	1.160475	.56	15.01
totalcalcium	9351	2.226458	.2850475	1.07	3.47
po4	9351	1.865383	.6744759	.4	5.83

hb	9351	96.37526	21.77429	10	187
pre_d_wt	9351	62.36245	12.1726	21.3	138.4
creat	9351	902.3062	305.5148	104	2471
KtV	9351	1.309344	.348188	.4	2.96
per	9351	3.227478	1.891966	-11.58392	11.20944

m=3 data:
-> **summ bmi alb tot_chol totalcalcium po4 hb pre_d_wt creat KtV per**

Variable	Obs	Mean	Std. Dev.	Min	Max
bmi	9351	22.55779	3.693098	10.47	46.02
alb	9351	35.80256	6.422867	11	57
tot_chol	9351	4.112422	1.155712	.56	15.01
totalcalcium	9351	2.225774	.2839098	1.07	3.47
po4	9351	1.86321	.6740408	.4	5.83
hb	9351	96.38192	21.73861	10	187
pre_d_wt	9351	62.35496	12.22204	21.3	138.4
creat	9351	901.9314	305.8412	104	2471
KtV	9351	1.307503	.3515729	.4	2.96
per	9351	3.237585	1.881957	-11.58392	11.20944

m=4 data:
-> **summ bmi alb tot_chol totalcalcium po4 hb pre_d_wt creat KtV per**

Variable	Obs	Mean	Std. Dev.	Min	Max
bmi	9351	22.5669	3.653953	10.47	46.02
alb	9351	35.77839	6.4074	11	57
tot_chol	9351	4.106101	1.154706	.56	15.01
totalcalcium	9351	2.228024	.2851763	1.07	3.47
po4	9351	1.865205	.6724103	.4	5.83
hb	9351	96.34704	21.90398	10	187
pre_d_wt	9351	62.42336	12.12095	21.3	138.4
creat	9351	904.4989	303.0878	104	2471
KtV	9351	1.31228	.350131	.4	2.96
per	9351	3.222661	1.883238	-11.58392	11.20944

m=5 data:
-> **summ bmi alb tot_chol totalcalcium po4 hb pre_d_wt creat KtV per**

Variable	Obs	Mean	Std. Dev.	Min	Max
bmi	9351	22.56642	3.678952	10.47	46.02
alb	9351	35.80102	6.405659	11	57
tot_chol	9351	4.110641	1.160171	.56	15.01
totalcalcium	9351	2.227844	.2851003	1.07	3.47
po4	9351	1.863443	.6729622	.4	5.83
hb	9351	96.33983	21.82927	10	187

pre_d_wt	9351	62.37936	12.19406	21.3	138.4
creat	9351	904.0697	305.4299	104	2471
KtV	9351	1.312621	.3497305	.4	2.96
per	9351	3.235625	1.896368	-11.58392	11.20944

m=6 data:
-> **summ bmi alb tot_chol totalcalcium po4 hb pre_d_wt creat KtV per**

Variable	Obs	Mean	Std. Dev.	Min	Max
bmi	9351	22.54483	3.686111	10.47	46.02
alb	9351	35.83733	6.448454	11	57
tot_chol	9351	4.104771	1.153079	.56	15.01
totalcalcium	9351	2.226039	.2844286	1.07	3.47
po4	9351	1.863208	.6763725	.4	5.83
hb	9351	96.31957	21.85859	10	187
pre_d_wt	9351	62.24581	12.16774	21.3	138.4
creat	9351	898.1337	300.2103	104	2471
KtV	9351	1.313078	.3485768	.4	2.96
per	9351	3.248965	1.889511	-11.58392	11.20944

m=7 data:
-> **summ bmi alb tot_chol totalcalcium po4 hb pre_d_wt creat KtV per**

Variable	Obs	Mean	Std. Dev.	Min	Max
bmi	9351	22.53272	3.677953	10.47	46.02
alb	9351	35.75447	6.440467	11	57
tot_chol	9351	4.105849	1.148643	.56	15.01
totalcalcium	9351	2.226259	.2851009	1.07	3.47
po4	9351	1.867211	.6718863	.4	5.83
hb	9351	96.52859	21.7677	10	187
pre_d_wt	9351	62.25696	12.09774	21.3	138.4
creat	9351	899.6177	301.664	104	2471
KtV	9351	1.31498	.3498772	.4	2.96
per	9351	3.234503	1.883772	-11.58392	11.20944

m=8 data:
-> **summ bmi alb tot_chol totalcalcium po4 hb pre_d_wt creat KtV per**

Variable	Obs	Mean	Std. Dev.	Min	Max
bmi	9351	22.55223	3.686109	10.47	46.02
alb	9351	35.80606	6.438991	11	57
tot_chol	9351	4.100273	1.155803	.56	15.01
totalcalcium	9351	2.226535	.2853106	1.07	3.47
po4	9351	1.868078	.6736903	.4	5.83
hb	9351	96.32368	21.78578	10	187
pre_d_wt	9351	62.32573	12.19885	21.3	138.4
creat	9351	899.6589	304.6795	104	2471

Variable	Obs	Mean	Std. Dev.	Min	Max
KtV	9351	1.318117	.3519594	.4	2.96
per	9351	3.250323	1.87169	-11.58392	11.20944

m=9 data:

-> summ bmi alb tot_chol totalcalcium po4 hb pre_d_wt creat KtV per

Variable	Obs	Mean	Std. Dev.	Min	Max
bmi	9351	22.52545	3.68337	10.47	46.02
alb	9351	35.82099	6.458985	11	57
tot_chol	9351	4.098799	1.151762	.56	15.01
totalcalcium	9351	2.226636	.2844736	1.07	3.47
po4	9351	1.869351	.6749128	.4	5.83
hb	9351	96.25351	21.90073	10	187
pre_d_wt	9351	62.24626	12.17812	21.3	138.4
creat	9351	891.8691	307.1837	104	2471
KtV	9351	1.302963	.3512762	.4	2.96
per	9351	3.239481	1.890999	-11.58392	11.20944

m=10 data:

-> summ bmi alb tot_chol totalcalcium po4 hb pre_d_wt creat KtV per

Variable	Obs	Mean	Std. Dev.	Min	Max
bmi	9351	22.50153	3.666376	10.47	46.02
alb	9351	35.76271	6.447166	11	57
tot_chol	9351	4.112494	1.157818	.56	15.01
totalcalcium	9351	2.225486	.2851898	1.07	3.47
po4	9351	1.865649	.6765171	.4	5.83
hb	9351	96.38995	21.90939	10	187
pre_d_wt	9351	62.21339	12.14194	21.3	138.4
creat	9351	900.622	304.3301	104	2471
KtV	9351	1.3107	.3561082	.4	2.96
per	9351	3.256491	1.889033	-11.58392	11.20944

m=11 data:

-> summ bmi alb tot_chol totalcalcium po4 hb pre_d_wt creat KtV per

Variable	Obs	Mean	Std. Dev.	Min	Max
bmi	9351	22.57419	3.652075	10.47	46.02
alb	9351	35.82146	6.400494	11	57
tot_chol	9351	4.120359	1.155184	.56	15.01
totalcalcium	9351	2.226003	.2834779	1.07	3.47
po4	9351	1.86933	.6748425	.4	5.83
hb	9351	96.40662	21.71819	10	187
pre_d_wt	9351	62.37766	12.20578	21.3	138.4
creat	9351	898.0257	308.5187	104	2471
KtV	9351	1.310586	.3500207	.4	2.96
per	9351	3.237837	1.898509	-11.58392	11.20944

m=12 data:
-> **summ bmi alb tot_chol totalcalcium po4 hb pre_d_wt creat KtV per**

Variable	Obs	Mean	Std. Dev.	Min	Max
bmi	9351	22.56545	3.658881	10.47	46.02
alb	9351	35.776	6.454754	11	57
tot_chol	9351	4.105501	1.153238	.56	15.01
totalcalcium	9351	2.226091	.2830665	1.07	3.47
po4	9351	1.865024	.6750385	.4	5.83
hb	9351	96.36171	21.77535	10	187
pre_d_wt	9351	62.34121	12.16249	21.3	138.4
creat	9351	895.1562	305.0878	104	2471
KtV	9351	1.312217	.3515409	.4	2.96
per	9351	3.242331	1.89596	-11.58392	11.20944

m=13 data:
-> **summ bmi alb tot_chol totalcalcium po4 hb pre_d_wt creat KtV per**

Variable	Obs	Mean	Std. Dev.	Min	Max
bmi	9351	22.56779	3.681538	10.47	46.02
alb	9351	35.7944	6.42771	11	57
tot_chol	9351	4.098636	1.143699	.56	15.01
totalcalcium	9351	2.226474	.2849409	1.07	3.47
po4	9351	1.859603	.6732934	.4	5.83
hb	9351	96.29529	21.82028	10	187
pre_d_wt	9351	62.32793	12.14438	21.3	138.4
creat	9351	899.4627	303.2246	104	2471
KtV	9351	1.315179	.3505051	.4	2.96
per	9351	3.25002	1.875526	-11.58392	11.20944

m=14 data:
-> **summ bmi alb tot_chol totalcalcium po4 hb pre_d_wt creat KtV per**

Variable	Obs	Mean	Std. Dev.	Min	Max
bmi	9351	22.53021	3.689616	10.47	46.02
alb	9351	35.80452	6.432571	11	57
tot_chol	9351	4.106324	1.150554	.56	15.01
totalcalcium	9351	2.225081	.2842888	1.07	3.47
po4	9351	1.862754	.6728443	.4	5.83
hb	9351	96.42768	21.76221	10	187
pre_d_wt	9351	62.24457	12.18611	21.3	138.4
creat	9351	904.6246	303.5673	104	2471
KtV	9351	1.311751	.3492853	.4	2.96
per	9351	3.255647	1.886973	-11.58392	11.20944

m=15 data:

-> **summ bmi alb tot_chol totalcalcium po4 hb pre_d_wt creat KtV per**

Variable	Obs	Mean	Std. Dev.	Min	Max
bmi	9351	22.53629	3.644038	10.47	46.02
alb	9351	35.79241	6.431524	11	57
tot_chol	9351	4.11328	1.147397	.56	15.01
totalcalcium	9351	2.226404	.2851723	1.07	3.47
po4	9351	1.863868	.6765802	.4	5.83
hb	9351	96.44574	21.81301	10	187
pre_d_wt	9351	62.2651	12.09914	21.3	138.4
creat	9351	898.6741	303.8313	104	2471
KtV	9351	1.311039	.3520433	.4	2.96
per	9351	3.227893	1.882465	-11.58392	11.20944

m=16 data:
-> **summ bmi alb tot_chol totalcalcium po4 hb pre_d_wt creat KtV per**

Variable	Obs	Mean	Std. Dev.	Min	Max
bmi	9351	22.54433	3.708164	10.47	46.02
alb	9351	35.8029	6.42912	11	57
tot_chol	9351	4.109556	1.151241	.56	15.01
totalcalcium	9351	2.228944	.2836	1.07	3.47
po4	9351	1.864969	.6743188	.4	5.83
hb	9351	96.30773	21.77041	10	187
pre_d_wt	9351	62.30401	12.18934	21.3	138.4
creat	9351	898.9772	305.3022	104	2471
KtV	9351	1.316223	.3471549	.4	2.96
per	9351	3.239908	1.880549	-11.58392	11.20944

m=17 data:
-> **summ bmi alb tot_chol totalcalcium po4 hb pre_d_wt creat KtV per**

Variable	Obs	Mean	Std. Dev.	Min	Max
bmi	9351	22.54163	3.674451	10.47	46.02
alb	9351	35.77254	6.452571	11	57
tot_chol	9351	4.112021	1.152516	.56	15.01
totalcalcium	9351	2.226579	.2841769	1.07	3.47
po4	9351	1.862923	.6710306	.4	5.83
hb	9351	96.36482	21.86602	10	187
pre_d_wt	9351	62.32854	12.17143	21.3	138.4
creat	9351	903.1272	304.6748	104	2471
KtV	9351	1.309188	.3473194	.4	2.96
per	9351	3.237986	1.892096	-11.58392	11.20944

m=18 data:
-> **summ bmi alb tot_chol totalcalcium po4 hb pre_d_wt creat KtV per**

Variable	Obs	Mean	Std. Dev.	Min	Max
bmi	9351	22.54871	3.643742	10.47	46.02
alb	9351	35.79393	6.425428	11	57
tot_chol	9351	4.095178	1.144835	.56	15.01
totalcalcium	9351	2.225549	.2844612	1.07	3.47
po4	9351	1.864318	.6789322	.4	5.83
hb	9351	96.3339	21.94124	10	187
pre_d_wt	9351	62.30692	12.11635	21.3	138.4
creat	9351	898.4123	304.5351	104	2471
KtV	9351	1.312685	.3493088	.4	2.96
per	9351	3.228534	1.879992	-11.58392	11.20944

m=19 data:
-> **summ bmi alb tot_chol totalcalcium po4 hb pre_d_wt creat KtV per**

Variable	Obs	Mean	Std. Dev.	Min	Max
bmi	9351	22.5267	3.674979	10.47	46.02
alb	9351	35.7512	6.437232	11	57
tot_chol	9351	4.106363	1.152432	.56	15.01
totalcalcium	9351	2.226478	.2848589	1.07	3.47
po4	9351	1.861935	.6750283	.4	5.83
hb	9351	96.34514	21.80529	10	187
pre_d_wt	9351	62.30696	12.20241	21.3	138.4
creat	9351	896.7303	304.8803	104	2471
KtV	9351	1.311813	.3504727	.4	2.96
per	9351	3.229612	1.879888	-11.58392	11.20944

m=20 data:
-> **summ bmi alb tot_chol totalcalcium po4 hb pre_d_wt creat KtV per**

Variable	Obs	Mean	Std. Dev.	Min	Max
bmi	9351	22.59233	3.7053	10.47	46.02
alb	9351	35.80153	6.420536	11	57
tot_chol	9351	4.10609	1.143563	.56	15.01
totalcalcium	9351	2.225865	.2847843	1.07	3.47
po4	9351	1.861117	.6741085	.4	5.83
hb	9351	96.31962	21.73306	10	187
pre_d_wt	9351	62.39721	12.17446	21.3	138.4
creat	9351	899.0332	305.6105	104	2471
KtV	9351	1.321002	.3524972	.4	2.96
per	9351	3.228285	1.887773	-11.58392	11.20944

```
3 .
4 . mi xeq: tab VA
```

m=0 data:
-> **tab VA**

va	Freq.	Percent	Cum.
AV Fistula/Graft {it:(Ref)}	4,653	52.67	52.67
CVC/other	4,182	47.33	100.00
Total	8,835	100.00	

m=1 data:
-> **tab VA**

va	Freq.	Percent	Cum.
AV Fistula/Graft {it:(Ref)}	4,904	52.44	52.44
CVC/other	4,447	47.56	100.00
Total	9,351	100.00	

m=2 data:
-> **tab VA**

va	Freq.	Percent	Cum.
AV Fistula/Graft {it:(Ref)}	4,925	52.67	52.67
CVC/other	4,426	47.33	100.00
Total	9,351	100.00	

m=3 data:
-> **tab VA**

va	Freq.	Percent	Cum.
AV Fistula/Graft {it:(Ref)}	4,919	52.60	52.60
CVC/other	4,432	47.40	100.00
Total	9,351	100.00	

m=4 data:
-> **tab VA**

va	Freq.	Percent	Cum.
AV Fistula/Graft {it:(Ref)}	4,919	52.60	52.60
CVC/other	4,432	47.40	100.00
Total	9,351	100.00	

m=5 data:
-> **tab VA**

va	Freq.	Percent	Cum.

AV Fistula/Graft {it:(Ref)}	**4,926**	**52.68**	**52.68**
CVC/other	**4,425**	**47.32**	**100.00**
Total	**9,351**	**100.00**	

m=6 data:
-> **tab VA**

va	Freq.	Percent	Cum.
AV Fistula/Graft {it:(Ref)}	**4,936**	**52.79**	**52.79**
CVC/other	**4,415**	**47.21**	**100.00**
Total	**9,351**	**100.00**	

m=7 data:
-> **tab VA**

va	Freq.	Percent	Cum.
AV Fistula/Graft {it:(Ref)}	**4,919**	**52.60**	**52.60**
CVC/other	**4,432**	**47.40**	**100.00**
Total	**9,351**	**100.00**	

m=8 data:
-> **tab VA**

va	Freq.	Percent	Cum.
AV Fistula/Graft {it:(Ref)}	**4,924**	**52.66**	**52.66**
CVC/other	**4,427**	**47.34**	**100.00**
Total	**9,351**	**100.00**	

m=9 data:
-> **tab VA**

va	Freq.	Percent	Cum.
AV Fistula/Graft {it:(Ref)}	**4,929**	**52.71**	**52.71**
CVC/other	**4,422**	**47.29**	**100.00**
Total	**9,351**	**100.00**	

m=10 data:
-> **tab VA**

va	Freq.	Percent	Cum.
AV Fistula/Graft {it:(Ref)}	**4,929**	**52.71**	**52.71**
CVC/other	**4,422**	**47.29**	**100.00**

Total	9,351	100.00

m=11 data:
-> **tab VA**

va	Freq.	Percent	Cum.
AV Fistula/Graft {it:(Ref)}	4,923	52.65	52.65
CVC/other	4,428	47.35	100.00
Total	9,351	100.00	

m=12 data:
-> **tab VA**

va	Freq.	Percent	Cum.
AV Fistula/Graft {it:(Ref)}	4,914	52.55	52.55
CVC/other	4,437	47.45	100.00
Total	9,351	100.00	

m=13 data:
-> **tab VA**

va	Freq.	Percent	Cum.
AV Fistula/Graft {it:(Ref)}	4,921	52.63	52.63
CVC/other	4,430	47.37	100.00
Total	9,351	100.00	

m=14 data:
-> **tab VA**

va	Freq.	Percent	Cum.
AV Fistula/Graft {it:(Ref)}	4,927	52.69	52.69
CVC/other	4,424	47.31	100.00
Total	9,351	100.00	

m=15 data:
-> **tab VA**

va	Freq.	Percent	Cum.
AV Fistula/Graft {it:(Ref)}	4,952	52.96	52.96
CVC/other	4,399	47.04	100.00
Total	9,351	100.00	

m=16 data:

-> **tab VA**

va	Freq.	Percent	Cum.
AV Fistula/Graft {it:(Ref)}	**4,934**	**52.76**	**52.76**
CVC/other	**4,417**	**47.24**	**100.00**
Total	**9,351**	**100.00**	

m=17 data:
-> **tab VA**

va	Freq.	Percent	Cum.
AV Fistula/Graft {it:(Ref)}	**4,917**	**52.58**	**52.58**
CVC/other	**4,434**	**47.42**	**100.00**
Total	**9,351**	**100.00**	

m=18 data:
-> **tab VA**

va	Freq.	Percent	Cum.
AV Fistula/Graft {it:(Ref)}	**4,904**	**52.44**	**52.44**
CVC/other	**4,447**	**47.56**	**100.00**
Total	**9,351**	**100.00**	

m=19 data:
-> **tab VA**

va	Freq.	Percent	Cum.
AV Fistula/Graft {it:(Ref)}	**4,935**	**52.78**	**52.78**
CVC/other	**4,416**	**47.22**	**100.00**
Total	**9,351**	**100.00**	

m=20 data:
-> **tab VA**

va	Freq.	Percent	Cum.
AV Fistula/Graft {it:(Ref)}	**4,934**	**52.76**	**52.76**
CVC/other	**4,417**	**47.24**	**100.00**
Total	**9,351**	**100.00**	

5 .
6 . log close
name: **<unnamed>**
log: **/Users/markmarshall/Dropbox/SRRN/Manuscript/appendix1.smcl**

```
  log type:  smcl
 closed on:  24 Jul 2015, 11:15:17
```
