# Supplementary material for: Outcomes and practice patterns with hemodiafiltration in Shanghai: a longitudinal cohort study
Source: BMC Nephrol. 2019 Feb 1;20:34. doi: 10.1186/s12882-019-1219-z (PMC6359843; doi:10.1186/s12882-019-1219-z)

Figure S2: Full competing risks main-effects model, fully adjusted for the main effects confounders listed in Table 1 (the marker represents point estimates, the whiskers, 95% confidence intervals). Abbreviations: HDF, hemodiafiltration; HB, hemoglobin; BMI, body mass index; eGFR, estimated glomerular filtration rate; RRT, renal replacement therapy; AV, arteriovenous; IDWG, inter-dialytic weight gain.

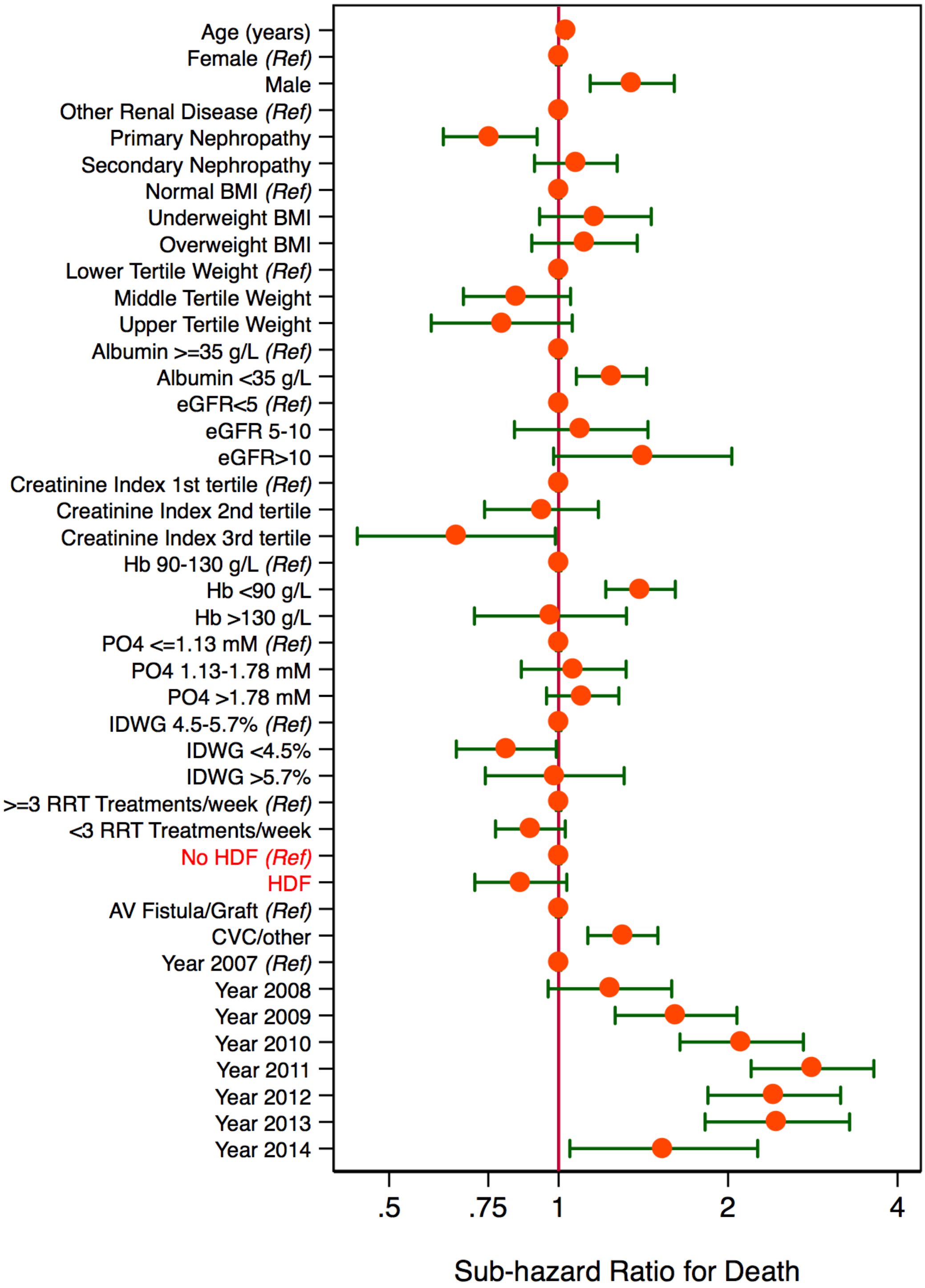

Supplement: Supplementary file 4 — Figure S2. Full competing risks main-effects model, fully adjusted for the main effects confounders listed in Table 1 (the marker represents point estimates, the whiskers, 95% confidence intervals). Abbreviations: HDF, hemodiafiltration; HB, hemoglobin; BMI, body mass index; eGFR, estimated glomerular filtration rate; RRT, renal replacement therapy; AV, arteriovenous; IDWG, inter-dialytic weight gain. (PDF 283 kb) [file 12882_2019_1219_MOESM4_ESM.pdf]
